# Supplementary material for: Characterisation of peri-implantation endometrial Treg and identification of an altered phenotype in recurrent pregnancy loss
Source: Mucosal Immunol. 2021 Sep 22;15(1):120–9. doi: 10.1038/s41385-021-00451-1 (PMC8732268; doi:10.1038/s41385-021-00451-1)
Supplement: Supplementary file 1 — Supplementary figures [file 41385_2021_451_MOESM1_ESM.pdf]

a

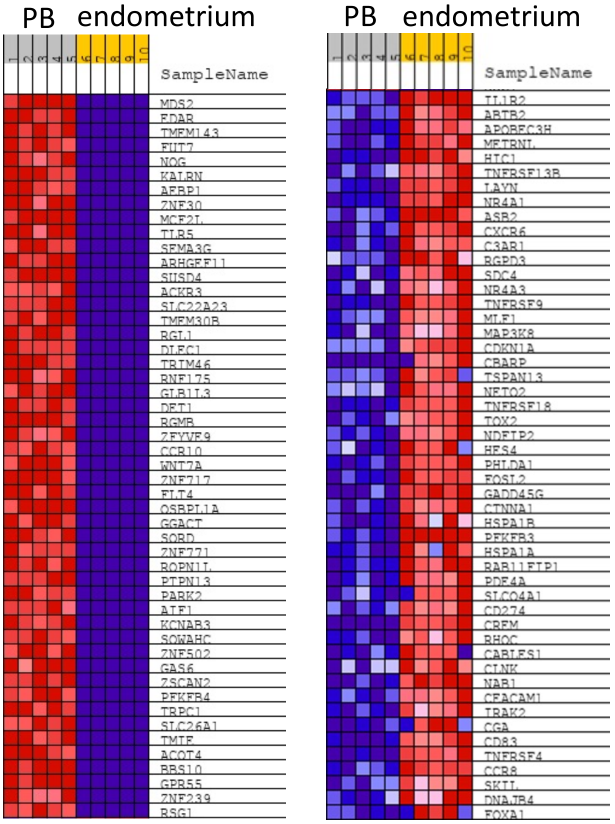

b

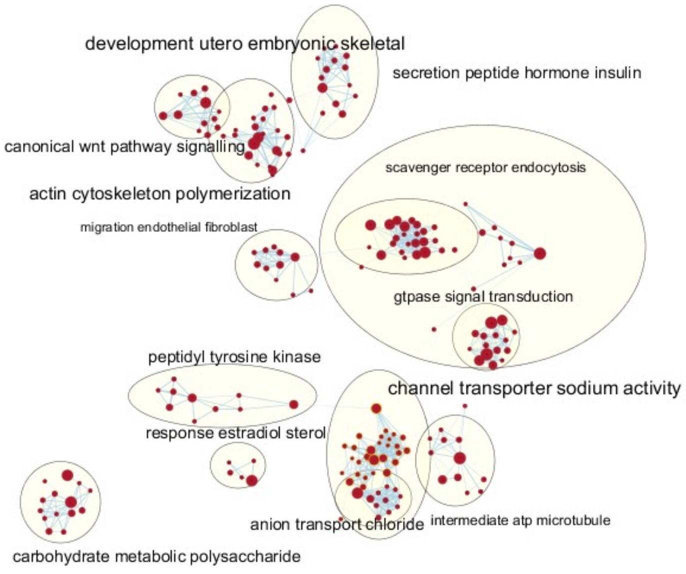

Supplementary Figure 1

Gene Set Enrichment Analysis (GSEA)(Broad Institute), was performed using the RNA transcriptome from peripheral blood (grey, 1-5) or endometrial (orange, 6-10) Treg. The Gene Ontology gene set database C5.all.v7.4 was used with 1000 permutations, leading edge analysis performed and Enrichment Map calculated with P-value cutoff 0.0005 and FDR Q-value cutoff 0.1, figures were visualized using Cytoscape 3.7.2 with ≥5 nodes selected. a) heatmap of top 50 altered genes between groups (red – high expression, blue – low expression), b) pathways enrichment map.

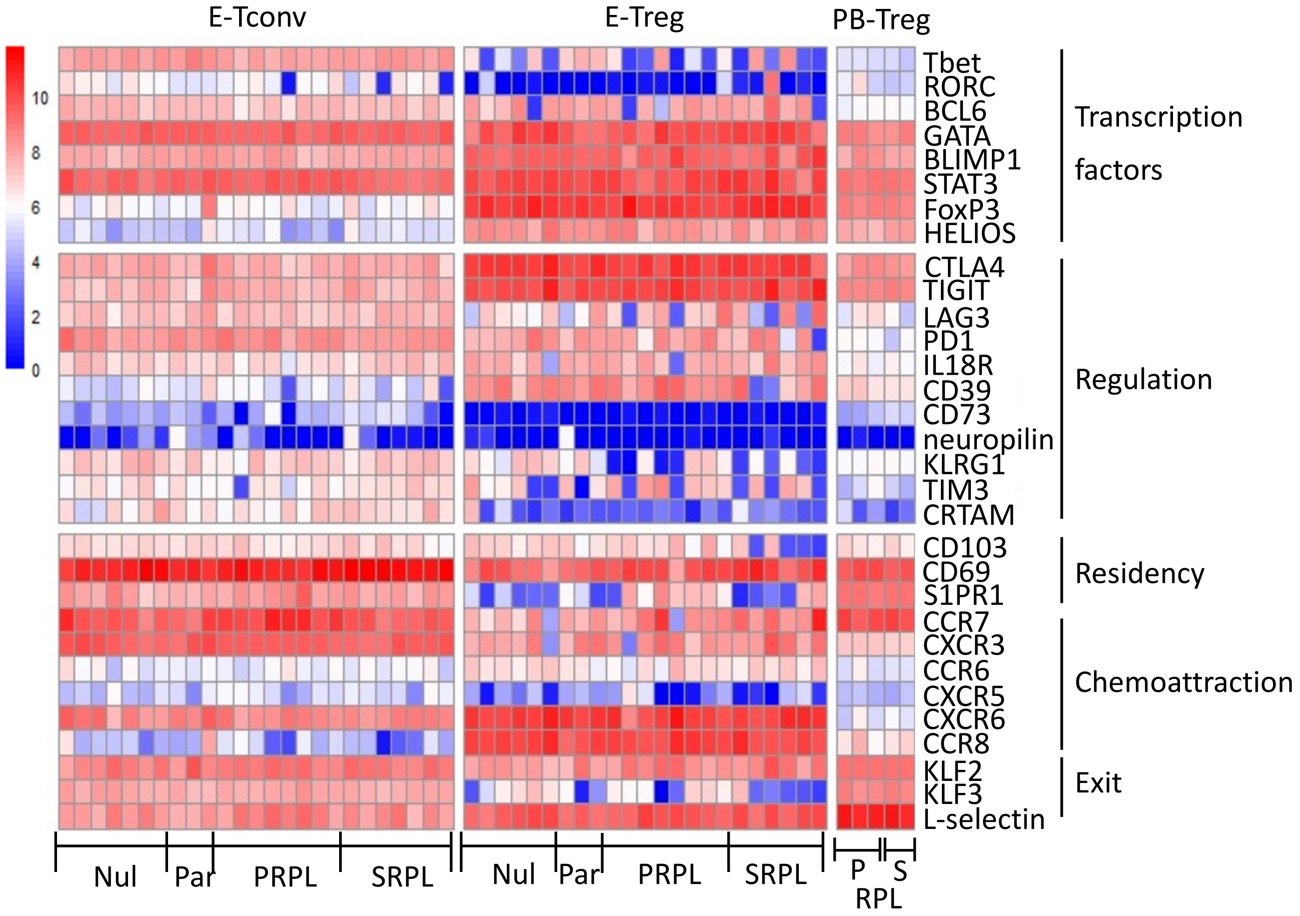

Supplementary Figure 2

Transcriptomic analysis of Treg derived from matched endometrium Tconv (E-Tconv) and Treg (E-Treg) (n=25) versus peripheral blood RPL Treg (PB-Treg) (n=5) from Nulluparous (Nul), Parous (Par), Primary RPL (PRPL) and Secondary RPL (SRPL) patients, heatmap of gene expression levels of known Tconv and Treg factors, red = high, blue = low expression
